# The Nigeria wealth distribution and health seeking behaviour: evidence from the 2012 national HIV/AIDS and reproductive health survey

**DOI:** 10.1186/s13561-015-0043-9

**Published:** 2015-02-11

**Authors:** Adeniyi F Fagbamigbe, Elijah A Bamgboye, Bidemi O Yusuf, Joshua O Akinyemi, Bolakale K Issa, Evelyn Ngige, Perpetua Amida, Adebobola Bashorun, Emmanuel Abatta

**Affiliations:** 1 Department of Epidemiology and Medical Statistics, Faculty of Public Health, College of Medicine, University of Ibadan, Ibadan, Nigeria; 2 NASCP, Federal Ministry of Health, Abuja, Nigeria

**Keywords:** Nigeria, Principal component analysis, Wealth index, Household asset, Health seeking behaviour

## Abstract

**Background:**

Recently, Nigeria emerged as the largest economy in Africa and the 26th in the world. However, a pertinent question is how this new economic status has impacted on the wealth and health of her citizens. There is a dearth of empirical study on the wealth distribution in Nigeria which could be important in explaining the general disparities in their health seeking behavior. An adequate knowledge of Nigeria wealth distribution will no doubt inform policy makers in their decision making to improve the quality of life of Nigerians.

**Method:**

This study is a retrospective analysis of the assets of household in Nigeria collected during the 2012 National HIV/AIDS and Reproductive Health Survey (NARHS Plus 2). We used the principal component analysis methods to construct wealth quintiles across households in Nigeria. At 5% significance level, we used ANOVA to determine differences in some health outcomes across the WQs and chi-square test to assess association between WQs and some reproductive health seeking behaviours.

**Result:**

The wealth quintiles were found to be internally valid and coherent. However, there is a wide gap in the reproductive health seeking behavior of household members across the wealth quintiles with members of households in lower quintiles having lesser likelihood (33.0%) to receive antenatal care than among those in the highest quintiles (91.9%). While only 3% were currently using modern contraceptives in the lowest wealth quintile, it was 17.4% among the highest wealth quintile (p < 0.05).

**Conclusion:**

The wealth quintiles showed a great disparity in the standard of living of Nigerian households across geo-political zones, states and rural–urban locations which had greatly influenced household health seeking behavior.

## Background

Peoples’ health and wealth status are closely related and have a dual-way relationship. A previously financial buoyant individual may be impoverished by ill-health, and poor health may arise from being poor if an individual is unable to afford adequate basic necessities such as sanitation, health care, food, and housing. A poverty-related lifestyle would ultimately reduce access to, and utilization of, health facilities and services [1-4].

Wealth is the aggregated values of all natural, physical and financial assets owned by a household, after netting off its liabilities [5,6]. It signifies the economic status of individuals and families. However, wealth inequality has been reported across the globe [5-9]. Wealth inequality, also referred to as income inequality, is the disparities in the ownership of assets, wealth or income among individuals in a population. Economic theories have attempted to explain the causes of income inequality. The Neoclassical economists considered inequalities as a phenomenon that arose from differences in productivity whereby more productive people earn higher. They opined that rising inequalities are results of widened productivity gap between highly-paid professions and lower-paid professions [10]. The Marxian economists had attributed rising income inequality to an inherent feature of capitalism whereby human capital is being substituted by machines and technologies, thereby reducing cost and increasing profits of the capitalists. It also reduces wages, and throws people out of employment and into deeper poverty [11].

The Socialists also ascribed wide disparities in wealth and income to means of production being owned by private individuals [12] thereby impoverishing vast majorities earning mere wages. As alternative, the Marxists advocated communist society where there would be common ownership of the means of production, where each individual citizen would have free access to the articles of consumption and reduce dependency on others [13]. While meritocracy favors an eventual society where an individual’s success is a direct function of his merit, or contribution, the liberals relying on Keynesian macroeconomic policies, suggested that capitalism should be reformed and sustained. However, regardless of the merits of these economic theories, wide wealth inequalities affects health seeking behaviours of individuals and could pose great danger on their overall well-being [14].

It is therefore important to understand wealth distribution within populations when studying their utilization of health services. Wealth Index (WI) is one of the methods of evaluating wealth distribution. It is a composite index composed of key asset ownership or income and expenditure; often used as a proxy indicator of level of household wealth [15-19]. In economic literature, poverty, a WI level, is often measured by either incomes or expenditures or the balance between the two [16,18]. The WI has also been used to demonstrate the measure and distribution of economic status generally across the globe and in particular to explain equity differences in health outcomes and services within a country. A poor country would still have variations within itself and the relatively rich household in a poor country may be poor in absolute terms. Wealth Index is computed independent of demographic characteristics such as sex, age, education and residence [17,18,20-22]. An adequate knowledge of a country’s wealth index will provide useful information to guide policy makers as well as government officials in their decision making process as well as relevant information to academia and researchers [22,23].

**Figure 1:**
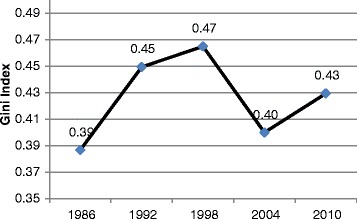
**Income inequality index in Nigeria.**

Alternatively, economic indices such as Gini coefficient derivable from Loren curve has been used to estimate disparities in income distributions among countries of the world. Gini index, developed in 1912, measures the extent to which income or consumptions among individuals or households within an economy deviate from a perfectly equal distribution. It relies on Lorenz curve which plots the cumulative percentages of total income received against the cumulative recipients, from poorest to richest categories. The Gini index is the ratio of area between the Lorenz curve and a hypothetical line of absolute equality and the maximum area under the line and ranges between 0.0 and 1.0 [7,8]. The closer the Gini Index is to 0 the lower the inequality. More developed countries have Gini coefficient of 0.20 to 0.30 while countries such as South Africa has about highest Gini index of 0.65 signifying high level of inequality [9]. Different Gini coefficients have been reported on Nigeria income distribution between 1986 and 2010. A Nigerian study which used a national data obtained Gini coefficients of 0.50, 0.53 and 0.60 for 1992, 1996 and 2000 respectively [7] compared to more modest estimates of 0.39, 0.45, 0.47, 0.40 and 0.43 for 1986, 1992, 1998, 2004 and 2010 respectively by the World Bank [9] as shown in Figure 1. All the coefficients showed that the Nigeria Gini index ranged between 0.39 and 0.6 implying a relatively high income inequality in Nigeria. According to the World Bank, Nigeria income distribution statistics between 1986 and 2010 showed that half of Nigeria income was earned by only highest 20% of the income earners peaking at 52.1% in 1998 while lowest 20% income earner gets only 5.0% of all incomes and about 10% earned by the next 20% [9].

There are renewed efforts to understand the distribution of wealth and poverty in Nigeria. For instance, a study aimed at explaining disparities in income distribution in Nigeria also used a Lorenz curve and Gini coefficient technique to estimate the effect of the new Federal Government salary scale on income distribution [8]. The study reported that the salary adjustment brought about reduction in inequality. Also a recent study on income inequality in Nigeria using the Lorenz curve and Gini Coefficient approach [7] found that Gini co-efficient of income inequality lied between 46 and 60 percent and attributed the inequalities to literacy. But, these efforts have been grossly undermined by unavailability of accurate and reliable data on the households income and expenditure in Nigeria [17-19,21,24]. The collection of accurate income or expenditure data in health-related household surveys is hampered by factors such as disclosure, seasonality, volatility, misreporting, and limited interview time [25]. Therefore, National health surveys do not routinely collect data on household expenditures but rather estimate relative wealth from household assets and housing characteristics [16].

Incidentally, several studies have found WI to be very useful in determining the likelihood of people to access health services especially when the services are not free. The positive influence of wealth on the use of health services has been corroborated by a Colombia study that reported wealthier mothers having higher likelihood of attending a first ANC visit and additional visits than poorer mothers [26]. Also, a Turkish study found that household wealth is positively and significantly associated with the choice of health facility for delivery [27].

Also, a Malian study on barriers to quality health utilization [28] reported that household poverty and personal problems are negatively related to the use of maternal health care. Similarly, in Ghana, the households in the wealthiest wealth quintile(WQ) are more likely to use antenatal services and deliver their babies in a health facility, than those in poorer and poorest WQs [29].

The 2008 and 2013 Nigeria Demographic and Health Surveys found fertility differentials by education and wealth. Women who have no formal education and women in the lowest WQ on average had 7 children, while women in the highest WQ with higher than a secondary education having 3 or 4 children. Also, women in the lowest WQs were more likely to have co-wives. Previous studies showed that Educational attainment was positively related to household wealth status. Women and men in the highest WQs were found to be more likely to be educated than those in the lowest WQs [30,31]. In the absence of relevant and reliable information on income and expenditure of households in demographic and health surveys, alternatives like the wealth index is used to estimate and comprehend the economic status of households based on asset possessions. Such yardsticks are also used for understanding poor-rich inequalities in demographic and health outcomes [31,32]. The 2012 National HIV and AIDS and Reproductive Health Survey (NARHS Plus) collected data on household assets or possessions such as livestock, tractor, plough, radio, refrigerator, television, bicycle, motorbike, phone/cell phone, chair, table, bed and households amenities such as water supply, toilet, flooring, walls/house, roof, electricity, cooking fuel and light source. Therefore, in this study the asset based approach was used to construct a wealth index across households in Nigeria using the NARHS Plus data. This was aimed at assessing disparities in the standard of living of Nigerian households vizaviz their health seeking behavior across geo-political zones, states and rural–urban location which could inform household abilities to access health care services.

## Method

This is a retrospective analysis of data from the 2012 National HIV/AIDS and Reproductive Health Survey (NARHS Plus) [33]. The NARHS Plus was a cross-sectional study of men and women of reproductive age. A stratified multistage cluster sampling technique was used to select a nationally representative probability sample of women aged 15–49 years and men aged 15–64 years living in households in rural and urban areas in all the 36 states and the Federal Capital Territory (FCT) Nigeria.

Stage 1 involved the selection of rural and urban localities from each state and FCT. Stage 2 involved the selection of Enumeration Areas (EA) within the selected rural and urban localities. Stage 3 involved the listing and selection of households. Thirty two households were sampled from each of the 30 sampled EA (clusters) from each state. Overall, 35,520 households were selected for final interview of which 32543 (91.6%) household heads or their proxies were successfully interviewed. However, only 30855 (86.9%) households provided valid information useful for this analysis.

Data was collected using two separate structured questionnaires; one for individual characteristics and the other for household assets and living conditions. The data was weighted to reflect differences in population sizes of the states.

### Construction of Wealth Index and Quintiles

Wealth Index was constructed using the asset approach whereby all household possessions are included as much as possible. They include the productive assets such as hand mill, sickle, axe, livestock, hoe, tractor, and plough; the non-productive assets such as radio, refrigerator, television, bicycle, motorbike, phone/cell phone, chair, table, and bed; the households amenities such as water supply, toilet, flooring, walls/house, roof, electricity, cooking fuel, and light source. The more the number of assets used in computing the wealth index the more its precision, accuracy and reliability [18]. Almost all household assets and utility services are to be included, including country-specific item because the greater the number of indicator variables, the better the representation of households.

Other alternative measures of economic status and poverty exist. They include the Gross National Income per capita based on purchasing power parity (GNI/p, PPP) [34]; Income approach to WI which was developed (Ferguson et al. 2003) for the World Health Survey (WHS) and Measures of Economic Status developed by the Institute for Health Metrics and Evaluation(IHME)and its modified version [20,35], International Wealth Index [23] and the Unsatisfied Basic Needs (UBN) approach [36]. The principal component analysis (PCA) method maintains as many variables as possible, theoretically relevant and has the advantage of avoiding the negative influence of high inter-correlation among variables [17].

The PCA used to assign the indicator weights for wealth index from the NARHS 2012 data following the UNICEF guidelines. This method has been described extensively in previous studies [21,24,37]. A Chinese study [4] which compared PCA with the principal axis factoring reported no significant difference in the outcomes of the two methods.

However, the PCA method of estimation of relative wealth is based on the first principal component and the WI for household *i* is the linear combination

1 $$ {\mathrm{y}}_{\mathrm{i}}={\upalpha}_1\frac{{\mathrm{x}}_1-{\upmu}_1}{{\mathrm{s}}_1}+{\upalpha}_2\frac{{\mathrm{x}}_2-{\upmu}_2}{{\mathrm{s}}_2}+\dots \dots \dots .+{\upalpha}_{\mathrm{k}}\frac{{\mathrm{x}}_{\mathrm{k}}-{\upmu}_{\mathrm{k}}}{{\mathrm{s}}_{\mathrm{k}}} $$

Where *μ*_*k*_ and *s*_*k*_ are the mean and standard deviation of asset *x*_*k*_ and *α* is the weight for each variable *x*_*k*_ for the first principal component.

The first principal component, either positive or negative, across households or individuals has a mean of zero and a variance of λ, which corresponds to the largest eigenvalue of the correlation matrix of *x.* The first principal component *y*_*i*_ yields a wealth index that assigns a larger weight to assets that vary the most across households so that an asset found in all households is given a weight of zero [24].

This procedure first standardizes the indicator variables (calculating z-scores); then the factor coefficient scores (factor loadings) were calculated; and finally, for each household (which an individual represents), the indicator values were multiplied by the loadings and summed to produce the household’s index value. In this process, only the first of the factors produced was used to represent the wealth index. The resulting sum is itself a standardized score with a mean of zero and a standard deviation of one.

2 $$ {\mathrm{q}}_{\mathrm{i}}=\mathrm{rank}\left\{\frac{{\mathrm{y}}_1}{n(5)}\right\} $$

The indicator variables are categorized and are broken into sets of dichotomous variables. For example, Radio: Yes = 1, No = 0. This forms an index having a weighted sum with an ad-hoc weighting scheme. The construction of the index requires several iterations before final results are obtained.

Rather than using percentiles for the wealth index, quintiles were used for easy comparison, whereby individuals were grouped into five equal groups based on their scores. Quintiles limit the number of categories to be tabulated and adequately represent the relationship between wealth and the phenomenon of interest. The households were then ordered by the score, and the distribution was divided at the points that form the five 20- percent distributions. Then the household score is recoded into the quintile variable so that each member of a household also receives that household’s quintile category. Following the categorization of the sampled households into 5 quintiles (*q*_*i*_) as shown in equation (2) where n(5) is the specification of equal groups desired, from the lowest to the highest. The PCA is usually used in measuring household socio-economic status because the index produces significant differences among different socio-economic groups, especially in the assets with high factor scores. Ordinarily, households in the higher and highest quintiles usually have the assets with high factor score and vice versa. We selected some of the assets used in the PCA irrespective of their factor score to assess the validity of the generated WQ from the wealth scores. The Predictive Analytics Software (PASW) 18 and STATA 12 were analysis. At 5% significance level, we used ANOVA to determine differences in some health outcomes across the WQs and chi-square test to assess association between WQs and some reproductive health seeking behaviours.

### Variables

In this study, we used a total of 32 indicator variables. They include toilet facilities, source of drinking water, type of housing, roofing materials used, floor materials used, wall materials used, house necessities such as radio, washing machine, television, mobile or fixed phones, mode of transportation (car, bicycle, motorcycle, donkeys), communications etc. We excluded variables with a prevalence below 3-5% or higher than 95-97% from the analysis so as to restrict computations to evenly distributed assets and to avoid assets owned and peculiar to only certain segments of the population.

## Results

Data were available from 30,855 households and their assets were used to divide the households into appropriate WQs. The result of the extraction of the principal components of the 32 assets variables in the data sets revealed there were 12 components with Eigen values higher than 1.0. These components jointly explained about 60% of variations in the data with component 1 explaining over 17% of the variance (Table 1).

**Table 1:** **Total variances explained in the principal factor analysis**

**Component**		**Initial eigenvalues**			**Extraction sums of squared loadings**			**Rotation sums of squared loadings**		
		**Total**	**% of variance**	**Cumulative %**	**Total**	**% of variance**	**Cumulative %**	**Total**	**% of variance**	**Cumulative %**
	1	5.572	17.413	17.413	5.572	17.413	17.413	4.764	14.888	14.888
	2	2.137	6.678	24.091	2.137	6.678	24.091	2.803	8.760	23.648
	3	1.477	4.616	28.707	1.477	4.616	28.707	1.331	4.160	27.808
	4	1.276	3.987	32.694	1.276	3.987	32.694	1.296	4.051	31.859
	5	1.243	3.885	36.579	1.243	3.885	36.579	1.258	3.931	35.790
	6	1.184	3.699	40.278	1.184	3.699	40.278	1.225	3.829	39.619
	7	1.077	3.366	43.644	1.077	3.366	43.644	1.133	3.539	43.158
	8	1.068	3.338	46.982	1.068	3.338	46.982	1.090	3.407	46.565
	9	1.042	3.257	50.239	1.042	3.257	50.239	1.073	3.354	49.919
	10	1.024	3.201	53.440	1.024	3.201	53.440	1.070	3.342	53.261
	11	1.016	3.176	56.616	1.016	3.176	56.616	1.061	3.316	56.578
	12	1.001	3.130	59.746	1.001	3.130	59.746	1.014	3.168	59.746
	13	.994	3.108	62.854						
	14	.954	2.980	65.834						
	15	.938	2.932	68.766						
	16	.916	2.861	71.627						
	17	.865	2.703	74.329						
	18	.851	2.658	76.988						
	19	.807	2.522	79.510						
	20	.748	2.339	81.849						
	21	.733	2.291	84.140						
	22	.706	2.205	86.345						
	23	.626	1.957	88.302						
	24	.601	1.878	90.180						
	25	.560	1.751	91.931						
	26	.556	1.738	93.669						
	27	.483	1.509	95.178						
	28	.404	1.264	96.442						
	29	.334	1.043	97.484						
	30	.282	.881	98.365						
	31	.280	.875	99.241						
	32	.243	.759	100.000						

Extraction Method: Principal Component Analysis.

**Figure 2:**
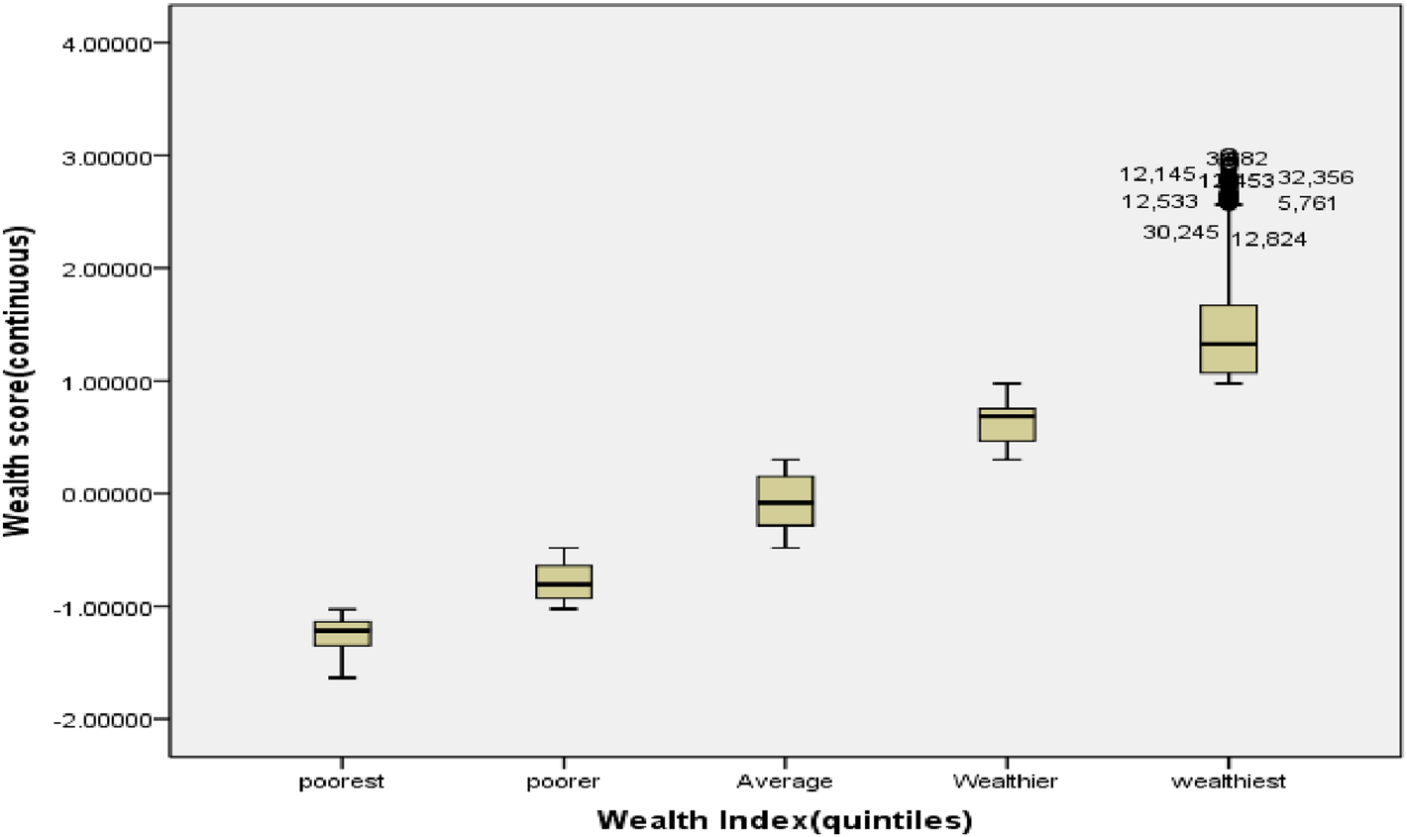
**Distribution of Wealth scores of individual households by Wealth Quintiles.**

The communalities or estimates of the variance in each variable accounted for by the components were high (Table 2). Also from the first principal component, having a television had the highest factor score (0.806) followed by having electric iron (0.771), electricity (0.707), a cement wall (0.696) and generating set (0.607). Figure 2 shows the distribution of the households into WQs as well as wealth scores and associated descriptive statistics for each quintile. The validity of the generated WQ from the wealth scores is as shown in Table 3. The percentage of households that possessed a television, cable TV, a tiled floor, an asbestos roof and a cement wall were very low in the two lowest quintiles. In fact less than 3% of households in the middle quintile and none in the lowest quintile possessed a cable TV. Almost 10% of households in the lowest quintile possessed radio and motorcycles and this increased to more than 20% in the middle quintile. The percentage of households with radio varied across the quintiles from lowest through highest quintile was 9.9% to 26.1%.

**Table 2:** **The component matrix with the wealth predictors**

	**Extractions**	**Component**											
		**1**	**2**	**3**	**4**	**5**	**6**	**7**	**8**	**9**	**10**	**11**	**12**
Electricity	.599	.707	-.247	-.109	-.014	-.057	-.074	.058	-.033	.080	.056	.056	-.003
Radio	.425	.435	-.174	.189	.294	-.038	.265	.057	.022	.020	.062	.041	.051
Television	.696	.806	-.152	-.071	.107	.051	.004	.017	-.043	.046	.013	.008	.011
Mobile telephone	.459	.595	-.257	.058	.167	.049	.045	-.012	-.032	-.037	.007	.006	-.028
Non-mobile telephone	.782	.134	.212	.104	-.162	-.003	.048	.221	.078	.516	.293	.469	.229
Refrigerator	.546	.664	.259	-.003	.011	.026	-.003	-.072	-.070	-.056	-.099	-.120	-.019
Cable TV/Network	.536	.526	.482	.126	-.072	.002	.015	-.052	-.011	.003	-.054	-.013	.023
Generating set	.479	.607	.154	.072	.111	.100	.078	-.068	-.001	-.147	-.108	-.110	-.055
Air-conditioner	.517	.337	.541	.182	-.209	-.041	.045	.072	.064	.069	.049	.110	.041
Computer/Laptop	.480	.419	.511	.110	-.135	-.056	.024	.012	.012	-.004	.034	.070	.059
Electric iron	.634	.771	.002	-.133	.114	-.019	-.020	-.013	-.048	.019	-.036	-.064	-.016
Fan	.705	.803	-.173	-.132	.097	.038	-.031	.008	-.025	.027	-.006	-.021	-.001
Canoe	.588	-.027	.044	-.056	-.004	.740	.088	-.060	.111	-.022	.083	-.048	-.031
Bicycle	.466	-.102	-.025	.380	.340	-.117	.358	.048	.203	-.008	.076	.052	.045
Motorcycle/Scooter	.475	.098	-.191	.392	.414	-.087	.305	-.005	.024	.008	-.017	.044	.026
Animal - Drawn cart	.224	-.222	.165	.122	.284	-.021	.195	-.091	.043	-.052	-.011	.002	.018
Car/Truck	.371	.425	.372	.126	-.033	-.060	.034	-.073	-.046	-.130	-.067	-.033	-.019
Boat with motor	.483	.040	.084	.004	.051	.517	.092	-.229	.263	.070	.264	.002	.014
Floor:Parquet	.508	-.001	.051	-.019	.083	.149	-.015	.628	-.259	.106	.009	-.024	-.050
Floor:Vinyl	.480	-.006	.031	.010	-.016	-.042	-.032	.127	.097	-.065	.438	-.334	.378
Floor:Tiles	.413	.295	.483	.155	-.092	-.029	-.032	-.019	-.004	-.126	-.054	-.136	-.147
Floor:Concr Cement	.810	.444	-.516	-.084	-.449	.022	.354	-.017	.029	-.042	-.004	.096	-.007
Floor:carpet	.842	.205	.049	-.106	.546	.016	-.650	-.055	.059	.215	.037	.033	.103
Roof:Zinc	.842	.344	-.413	.616	-.234	.127	-.296	-.019	-.115	.033	-.004	-.017	.012
Roof:wood	.546	-.058	.089	-.136	.121	.101	.159	.333	-.245	-.038	-.456	.186	.227
Roof: Calam/Cement	.750	.041	.061	-.094	.097	-.003	-.015	.078	.240	.121	.015	.316	-.740
Roof:tile	.661	.031	.054	-.077	.028	.017	.104	.500	.021	-.018	.286	-.472	-.291
Roof: concretecement	.906	.119	.053	-.387	-.012	-.091	.242	-.073	.385	.514	-.352	-.334	.135
Roof:asbestors	.866	.212	.122	-.592	.144	-.165	.152	-.079	-.095	-.374	.364	.295	.101
Wall:Cement or stone	.710	.696	-.284	-.086	-.169	-.039	-.115	.132	.253	-.086	-.011	.050	.044
Wall:brick	.919	.053	.014	-.071	.020	.010	.214	-.340	-.684	.438	.212	-.139	-.160
Wall:Wood planks/sph	.402	-.046	.073	-.093	.079	.536	.046	.077	-.131	-.096	-.168	.126	.121

Extraction Method: Principal Component Analysis with 12 components extracted.

**Table 3:** **Distribution of households with specific assets by wealth quintile**

**Variable (Asset)**	**Lowest**	**Lower**	**Middle**	**Higher**	**Highest**	**N**
Use electricity	0.9	8.8	24.5	31.6	34.1	17478
Radio	9.9	18.5	21.2	24.3	26.1	22626
Television	0.2	2.1	19.7	37.7	40.3	15200
Mobile phone	3.8	17.9	23.5	27.0	27.8	21854
Cable TV	0.0	0.1	2.9	9.1	87.9	2836
Motorcycle	10.7	22.2	22.1	23.8	21.2	10712
Car/Truck	1.1	5.3	6.3	13.3	74.0	2796
Floor: Tiles	0.0	1.7	6.3	13.6	78.5	966
Roof: asbestos	0.3	4.5	12.9	34.8	47.5	1508
Wall: Cement/ Stone block	0.2	8.5	24.2	31.8	35.2	16579

Over a quarter (27.5%) of households in the rural areas belonged to the lowest WQ compared with only 3.8% in urban areas. In North East and North West zones, about 41% households each belong to the lowest WQ compared with 32.1% among South West households in the highest WQ (Table 4).

**Table 4:** **Distribution of wealth quintiles by location and zones**

**Wealth quintiles**	**Lowest**	**Lower**	**Middle**	**Higher**	**Highest**	**N**
Location						
Rural	27.5	25.7	21.2	15.1	10.5	9782
Urban	3.8	7.7	17.5	30.6	40.3	21073
Zone						
North East	41.7	29.3	13.8	8.5	6.8	4673
North West	40.9	27.7	14.6	8.9	7.9	6179
North Central	16.0	21.8	21.3	19.0	21.9	5635
South East	6.9	13.3	26.2	27.6	26.0	4301
South South	5.6	13.3	26.0	27.5	27.8	4994
South West	4.4	12.3	19.8	31.5	32.1	5073

In Table 5, Yobe and Zamfara states had over 50% households in the lowest WQ when compared to other states with only 2.2% and 1.9% respectively making the highest WQ. The 10 states with highest proportion of households in the lowest WQ are from the Northern states of Nigeria (Table 5). Lagos state and the FCT topped the list of states with highest proportions of households belonging to the highest WQ with 58.1% and 55.1% respectively. Also more than a third of households in Rivers, Delta, Edo, Anambra and Abia states were in the highest WQ.

**Table 5:** **Distribution of wealth quintiles by states and the FCT**

	**Wealth quintiles**					
**State**	**Lowest**	**Lower**	**Middle**	**Higher**	**Highest**	**N**
Yobe	65.9	24.9	3.9	3.2	2.2	539
Zamfara	56.3	31.0	7.4	3.3	1.9	931
Sokoto	49.9	20.8	12.7	9.4	7.1	897
Borno	49.3	34.2	11.2	4.2	1.2	816
Kebbi	48.8	26.5	11.0	6.8	6.8	952
Jigawa	46.8	31.2	12.6	6.4	3.0	897
Taraba	45.9	28.6	12.0	6.2	7.3	936
Katsina	40.8	30.8	16.3	7.0	5.2	892
Gombe	36.7	27.9	15.6	11.2	8.6	795
Bauchi	34.6	26.9	18.9	11.9	7.6	866
Kano	31.3	23.2	18.3	12.5	14.7	672
Ebonyi	28.2	34.7	24.1	8.1	5.0	877
Benue	27.1	23.8	19.0	16.9	13.3	728
Adamawa	23.7	32.2	18.2	13.2	12.8	721
Nasarawa	23.4	37.0	25.8	8.2	5.6	765
Plateau	22.9	33.0	19.1	8.6	16.4	864
Niger	21.3	27.7	21.4	15.4	14.2	855
Kwara	12.3	10.2	18.8	31.1	27.5	861
Oyo	11.1	19.7	18.8	25.1	25.2	903
Kaduna	10.4	29.0	24.7	17.7	18.1	938
Bayelsa	8.4	19.5	26.2	25.1	20.8	821
Cross River	7.8	19.2	29.7	22.2	21.1	871
AkwaIbom	6.0	16.0	30.8	26.7	20.6	938
Rivers	5.9	8.7	19.7	29.0	36.7	645
Ondo	5.8	16.8	22.6	27.9	26.8	619
Kogi	4.2	15.0	30.3	27.2	23.3	838
Delta	4.0	7.4	24.2	28.0	36.3	900
Ogun	3.8	14.6	21.4	32.8	27.5	899
Ekiti	3.5	17.6	25.4	29.1	24.3	879
Enugu	3.3	16.4	29.9	22.7	27.7	669
Osun	2.4	4.3	21.2	42.1	30.0	921
Anambra	1.6	7.1	23.5	33.1	34.6	910
Edo	1.3	7.7	23.0	34.6	33.5	819
Abia	.8	6.9	26.7	31.6	34.2	919
FCT	.7	4.8	14.1	25.3	55.1	724
Imo	.4	3.5	27.6	40.5	28.0	926
Lagos	.0	1.6	10.0	30.3	58.1	852

Categorization of household’s reproductive health behavior by wealth status showed wide gaps between the rich and the poor as shown in Table 6. While 33.0% of individuals from households within the lowest quintile attended antenatal care service during the last pregnancy, 91.9% had it in the highest WQ just as 2.9% and 17.4% were currently using modern contraceptives in the lowest and highest WQ respectively. Conversely, members of households in lowest WQ had earlier sex debut than those from highest WQ (17 vs 22 years) and higher number of children ever born than households in higher WQs (4.6 vs 3.3 Children).

**Table 6:** **Reproductive health characteristics of respondents by wealth quintiles**

**Reproductive health characteristics**	**Lowest**	**Lower**	**Middle**	**Higher**	**Highest**
Had antenatal care during last pregnancy*	33.0	47.4	74.0	80.9	91.9
Used malaria drug during last pregnancy*	20.1	25.5	40.3	47.0	60.8
Can afford condoms*	43.4	55.4	64.2	72.8	76.7
Ever used male condom*	14.8	24.8	35.9	44.5	53.2
Currently using Modern Contraceptives*	2.9	5.9	10.6	14.6	17.4
Ever had a child*	75.7	74.2	66.4	64.6	61.4
Median age at first birth (years)	17	18	19	20	22
Children ever born**	4.6	4.5	4.1	3.7	3.3

*Significant at 5% in chi-square test. **Significant at 5% ANOVA.

## Discussion

The Nigeria wealth index developed in this study is internally valid and coherent as it shows a clear differentiation of living standards among different households. In fact, WI generated using the PCA, in measuring household socio-economic status has been found to produce significant differences among different socio-economic groups, especially in the assets with high factor scores. And ordinarily, households in the higher and highest quintiles usually have the assets with high factor score. The result further showed great disparity in living standards of Nigerian households across geo-political zones, states and rural–urban locations. This disparities would definitely affect households’ health seeking behaviors as further corroborated by previous studies [14,26-29,38].

Most households belonging to the highest quintiles possessed assets with high principal component factor scores just as those in lowest quintiles possessed only assets with low factor scores. These findings provide empirical evidence that the wealth index has the capability of providing a reliable and consistent means of measuring and ranking standard of living across all sectors and sections of Nigerian households. Despite the theoretical and practical advantage of the wealth index, it does not produce results that are similar to either an income- or expenditure-based index, [19] as compared to previous studies [25,39]. While Montgomery et al. (2000) noted that the WI may not be efficient as a proxy for measuring income distribution [25], Filmer and Pritchett (2001) concluded that WI had advantage of allowing better analysis of education differentials by economic status than did an expenditure index [39]. A major advantage of using a single distribution is that it eliminates confusion that multiple distributions may entail. However, the index may be weak in monitoring changes in poverty over time because there could be significant changes in household ownership of assets, which may not necessarily translate into poverty alleviation.

The use of these economic proxies (consumer durables, housing quality, household amenities and land holding size) collected to measure the economic status of the households in both small and large scale population-based surveys had been justified earlier [17,40,41]. The justification was based on the fact that it is easy to obtain information on economic proxies from households through simple questions or direct observation [40] rather than incomes which might have been falsified. Despite the popular use of assets, there are concerns about various issues such as the choice of indicators, the weight of individual indicators, the treatment of missing values and the choice of aggregation of indicators in the construction of composite indices [42,43]. We used a total of 32 assets in this analysis to arrive at the WQs. This number is quite higher than the eight assets used in a Mauritania study [15].

This study also showed that WI was sensitive to the types of each of the assets involved in the analysis. For example, assets such as motorcycle and radio which are owned by most households irrespective of living standards had nearly same proportions in the five quintiles. This distribution is typical of Nigerian socio-economic life. While nearly every household in the northern states of Nigeria possess the radio, the motorcycle is indeed the commonest means of transport in most of the states. This is very different from the distribution of some rare assets such as cars/trucks and the ownership of cable television which were essentially restricted to those in the highest quintiles. Also, the communalities or estimates of the variance in each variable accounted for by the components were high which indicates that the extracted components represent the variables well enough.

Our findings that the highest proportions of states in the Northern parts of Nigeria were in the two lowest quintiles is similar to the report by a McArthur foundation study [44] that reported the highest incidence of poverty as measured by Food-Energy household consumption in the three Northern zones of Nigeria. Poverty has been reported to be among known features peculiar to Northern Nigeria apart from early marriage and child bearing, low level of education, widespread poverty, gender disparity resulting in less woman autonomy and decision-making power, low utilization of healthcare service, high fertility and closely spaced birth interval [38]. The Northern zones in Nigeria, particularly the North East zone had most of its states’ households in the lowest WQ. Our finding is further corroborated by a UNDP report that cited huge regional differences in Nigeria human development index [45] which reported 0.332 for North East and 0.523 to the South West.

The large differences we found in wealth distributions in rural and urban Nigeria might further explain documented rural–urban differentials in utilization of health services such as antenatal care and use of modern contraceptives. We found higher likelihood of contraceptive and antenatal care services utilization among members of households in the higher WQs than among those in the lower while those in lower WQs also had higher tendency of early child bearing and larger families. This is in agreement with the outcomes of a Ghanaian study which found considerable variations in the use of antenatal care in the geographical regions and between the rural and urban dwellers and attributed this to differences in wealth status. The study recommended that “to improve the use of antenatal care and hence maternal health care utilization, some means of support should be provided especially to women within the lowest WQ, women should be encouraged to pursue education to at least the secondary level since this improves their use of maternal health services” [46].

Our study further showed that ownership of assets is suitable and appropriate to classify the households into WQs. This is similar to reports of a Mauritania study [15] where it was reported that “the rate of classification between using the index and household expenditure to identify the poor is relatively high. The huge disparities in the WI obtained in this study are in consonance with early findings that reported a relatively high Gini coefficient for Nigeria [7-9]. The reports showed that significant variations exists in income inequality and gaps between the rich and the poor in Nigeria.

## Conclusion

The wealth quintiles, a proxy for household wealth distribution, have shown the wealth status of Nigerian households and great disparities in the standard of living across geo-political zones, states and rural–urban locations. This huge differences in asset possession by households is a function of household’s income which affects their health seeking behavior [14,41]. There is need for Nigerian government and its supporting partners to develop policies aimed at improving asset possession of those in the lowest WI and also to put up accessible and affordable health care services to ensure the poor do not lack quality health care.

### Strengths and limitations of the study

Although the 32 assets used in this study to obtain wealth distribution in Nigeria are more than the eight assets used in a Mauritania study [15] and 15 and 11 assets used in the rural and peri-urban villages respectively in a Chinese study [4], inclusion of more assets to provide for regional differences in values and preferences would improve its accuracy. There was no variable in the data indicating household incomes; National surveys should include variables on household incomes so as to provide sufficient information for accurate estimation and further understanding of households’ wealth inequalities and health seeking patterns.
